# Frailty indexed classification of Bangladeshi older adults’ physio-psychosocial health and associated risk factors- a cross-sectional survey study

**DOI:** 10.1186/s12877-020-01970-5

**Published:** 2021-01-06

**Authors:** Mohammad Meshbahur Rahman, Mohammad Hamiduzzaman, Mst. Saleha Akter, Zaki Farhana, Mohammad Kamal Hossain, Mohammad Nayeem Hasan, Md. Nazrul Islam

**Affiliations:** 1 Biomedical Research Foundation, Dhaka, 1230 Bangladesh; 2 Basic Science Division, World University of Bangladesh, Dhaka, 1230 Bangladesh; 3 Flinders University Rural Health SA, College of Medicine & Public Health, Flinders University, Bedford, South Australia Australia; 4 Asian University of Bangladesh, Dhaka, Bangladesh; 5 Bangladesh Bank-The Central Bank of Bangladesh, Dhaka, 1215 Bangladesh; 6 Department of Statistics, Shahjalal University of Science and Technology, Sylhet, 3114 Bangladesh

**Keywords:** Older adults, Physical and mental health, Frailty index, Bangladesh

## Abstract

**Background:**

Frailty is associated with healthy ageing, and it has been identified as a means of measuring older adults’ physio-psychosocial health. We know about the ageing trends and common diseases of older adults living in South Asia, but literature to date does not widely feature their health status based on frailty, especially in Bangladesh. This study aims to understand the prevalence of frailty in Bangladeshi older adults; classify their health status; and investigate associated risk factors.

**Methods:**

A cross-sectional study was conducted in the north-eastern region (i.e. Sylhet City Corporation) of Bangladesh. Four hundred participants aged 55 years and above were randomly selected, attended a health assessment session and completed a multi-indicator survey questionnaire. We developed a 30-indicator Frailty Index (FI_30_) to assess the participant’s health status and categorized: good health (no-frailty/Fit); slightly poor health (mild frailty); poor health (moderate frailty); and very poor health (severe frailty). Pearson chi-square test and binary logistic regression analysis were conducted.

**Results:**

The participants’ mean age was 63.6 years, and 61.6% of them were assessed in poor to very poor health (moderate frailty/36.3% - severe frailty/25.3%). The eldest, female and participants from lower family income were found more frailty than their counterparts. Participants aged 70 years and above were more likely (adjusted OR: 4.23, 95% CI: 2.26–7.92, *p* < 0.0001) to experience frailty (medical conditions) than the pre-elderly age group (55–59 years). Female participants were more vulnerable (adjusted OR = 1.487, 95% CI: 0.84–2.64, *p* < 0.0174) to frailty (medical conditions) than male. Also, older adults who had higher family income (Income>$473.3) found a lower risk (adjusted OR: 0.294, 95% CI: 0.11–0.76, *p* < 0.011) of frailty (poor health).

**Conclusion:**

Our study results confirm the prevalence of frailty-related disorders in Bangladeshi older adults and highlight the importance of targeted clinical and community-led preventive care programs.

## Background

Increasing longevity comes with a high rate of morbidity, comorbidity and chronic conditions in South Asian older adults. The population of older adults is increasing in South Asia, and this steady increase is also evident in the change of life expectancy from 62.8 years in 2000 to 69.5 years in 2018 [1, 2]. Healthy ageing refers to an absence of physical and psychological conditions, but all South Asian countries ranked low in geriatric care, as such many older adults live with weakness, pain, obesity, eyesight, hearing loss, high blood pressure, diabetes, heart disease, and psychological problems [1]. These conditions are progressive in nature - require acute care needs – but are not being reported until they become severe. In India, Kashikar and Nagarkar (2016) reported the prevalence of frailty as 26% (Pre-frail- 63.6%; and Non-frail-10.4%) [3], while Ali et al. (2019) found frailty in 55.4% Pakistani older adults (Intermediate frail – 44.6%) [4]. In Nepal, the prevalence of frailty was 46.2% (men - 46.3%; women - 46.1%), and this prevalence was 15.2% in Sri Lanka (Pre-frail – 48.5%) [5]. The identified risk factors were level of education, occupation, older age, smoking, isolation, breathing problems, pain, fatigue, and falls and fractures [6]. Growing older population and their health conditions challenge public health leaders and clinicians in South Asia, especially in Bangladesh where geriatric care services and resources are limited.

In Bangladesh, the number of older adults has increased from 5.2 million in 1990 to 15 million in 2017, and about half of them experience the complications of malnourishment and diverse types of disability [1, 7, 8]. The literature indicates the older adults’ sedentary lifestyle that deteriorates their physical strength and intellectual capacity leading to an incompetence in daily living and a prevalence of injuries, falls and poor quality of life [2]. In addition, the prevalence of isolation, anxiety and depression are common in these older adults [7]. Their physical and mental health and health service utilization fluctuate based on socio-demographic structures and living circumstances [e.g. age, gender, education, occupation, income, lifestyle and family]. Fear of dependency on family members in the older adults because of illness is higher than their economic reliance [8, 9]. There is an increasing research interest on aging trends and diseases, but the elderly health status remains under-assessed in relation to their frailty, in turn best-practice was absent in making clinical and public health decisions for them.

Frailty is a state of vulnerability characterized by physio-psychosocial changes and loss of resistance to stressors caused by accumulated age-related deficits [10, 11]. Investigations on the older adults’ health relate the frailty with a risk of various negative health and well-being outcomes including physical weakness, falls, injuries, disability, poor quality of life, dementia, hospitalization, emergency department presentation and nursing home placement [12, 13]. As such, a Frailty Index approach is an effective means of elderly health measure [12, 13]. In Bangladesh, majority of the community-dwelling older adults have a tendency of self-care and avoiding hospitals, have health illiteracy, poverty lack of health services, and their frailty-related disorders remain undiagnosed and untreated. This unhealthy ageing causes a care and financial burden for the older adults and their family members, service providers and the community.

Studies are limited on older adult’s frailty in South Asia, and the available studies suggest contextual investigation of their frailty and health conditions [4, 14–20]. Few studies on ageing conducted in Bangladesh reported the importance of preventive and primary healthcare programs for older adults [7, 9, 21–28]. But having a lack of research on the older adults’ frailty and its associates caused an absence of evidence-based geriatric practice and service provision. Also, the studies conducted in Bangladesh did not investigate the country’s north-eastern population group, where most of the older adults presented comorbidity and multimorbidity [6]. We, therefore, investigate the prevalence of frailty, using a Frailty Index, in community-dwelling older adults in the north-eastern region of Bangladesh and classify their health status, and explore associated socio-demographic covariates.

## Methods

### Ethics and permission for data collection

Ethics approval for this study (Project Number: PS/2018/1/11) was granted by the University Research Center, Shahjalal University of Science & Technology, Bangladesh and formal permission for data collection was sought from Sylhet Civil Surgeon Office, Bangladesh.

### Study design

A cross-sectional study design, involving a person-centered general health assessment and a self-administered survey, was employed in this research.

### Setting and participants

This study was conducted from January 2018 to December 2019 in the north-eastern region (i.e. Sylhet City Corporation) of Bangladesh. In Bangladesh, while elderly cut-off age is 60 years and the national average of life expectancy is 72.05 years, literature notes that people who live in Sylhet have a lower life expectancy than the national average [29]. It is evident in literature that physical fitness or health condition of a person starts decline at the age of 55 years and present signs of aging (wrinkles, dullness of skin, weakness, dry skin, blotchiness and age spots) [30–34]. In Sylhet, the health literacy is low and the people are not aware of their healthcare needs until their problems are manifested [35]. As a result, we approached the people aged 55 years and above through Sylhet City Corporation. The inclusion criteria were: (i) aged 55 years and older; (ii) living in Sylhet City Corporation; and (iii) agreed to participate in a general health assessment and an interview. We used the single-stage cluster sampling, and this resulted in a random sample size of 400 participants from 13 administrative wards of the City Corporation. The administrative wards were selected through random number generated by R-Programming language.

### Data collection

A multi-indicator survey design was employed to explore diverse health issues and conditions of the participants. In accordance with the standards of Helsinki Declaration of 2000 (revised version), written informed consent was obtained from each of the participants. After formal consent, the health profile of each participant was collected, through a general health assessment and a structured questionnaire, including self-reported health problems, biomarkers, performance of daily activities and socio-demographic information. Necessary medical equipment was provided to a trained public health assistant to assess and collect anthropometric data.

### Outcome measures

#### General health assessment of height, weight, BMI, BP and RBS

The following Table 1 presents the measurement system of basic health indicators.

**Table 1:** Measurements of Height, Weight, BMI, BP and RBS

Health Indicators	Instruments	Measurement procedures
Height (m)	Height measuring scale (Stadiometer)	- Stand without shoes and simple summer clothes - Look straight ahead and keep shoulders to level
Weight (Kg)	Weight measuring scale (Seca Digital)	-Keeping normal Summer clothes - Keeping the respondents simple as far as possible during the measurement
BMI (Kg/m^2^)	Computer	$BMI=\frac{Weight(Kg)}{Height{(m)}^2}$
BP (mmHg.)	Electronic BP Monitor (OMB) Model: BP-1307	- Well seated - After 5 min rest - Average of three consecutive readings
RBS (mmol/L)	Digital RBS Machine (Vivacheck™ Ino.), Model: VGM01	- Time between breakfast and lunch - Time between lunch and dinner

#### Frailty index (FI_30_) measurement and coding of variables

Developing a list of variables is important to compute frailty, while there is no uniform list and researchers used different variables in calculating the Frailty Index. We consulted with clinicians and an epidemiologist, included 30 categorical variables specific to the older adults’ frailty and relevant to our socioeconomic contexts, following the works of Gobbens et al., 2010; Searle et al., 2008; and Rockwood et al., 2018 (Table 2) [36, 37]. Categorical variables were coded using the convention that ‘0’ indicated the absence of the deficit, and ‘1’ the presence of a deficit [36–38]. For some categorical variables (e.g. self-rated health, BMI etc.) that comprised one or more intermediate responses (e.g. ‘average’ or ‘frequently’), we considered the additional value of ‘0.25′, ‘0.5′ and ‘0.75′. We used the short form of Geriatric Depression Scale and a list of 15 questions in a separate questionnaire to collect data [39]. The mathematical formula used for calculating FI_30_ in this study was as follows:

1 $$ FI=\frac{\sum \mathrm{D} eficits}{30-\sum Missing\ Values} $$

**Table 2:** List of variables included in FI_30_

SN	Variable	Code (cut point)	SN	Variable	Code (cut point)
1.	Self-rated health status	[Good = 0, Average = 0.5, Poor Health = 1]	16.	Do you need help to carry more than 5 KG?	[No = 0, Yes = 1]
2.	Have any difficulties with hearing	[No = 0, Yes = 1]	17.	RBS	[0 (less than 7.9), 0.5 (7.9–14.9), 1 (15.0 or more)
3.	Do you have cataracts?	[No = 0, Yes = 1]	18.	Need any assistance when dressing?	[No = 0, Yes = 1]
4.	Have any difficulties with vision	[No = 0, Yes = 1]	19.	Do you need any stick for walking?	[No = 0, Yes = 1]
5.	Do you have anemia?	[No = 0, Yes = 1]	20.	Geriatric Depression Scale (GDS)	[Normal depression = 0, Moderate depression = 0.5, Severe depression =1]
6.	Do you have angina?	[No = 0, Yes = 1]	21.	Have you had heart attack?	[No = 0, Yes = 1]
7.	Do you have asthma?	[No = 0, Yes = 1]	22.	HBP	[Normal = 0, Mild = 0.33, Moderate = 0.66, Severe = 1]
8.	Balance (Do you need assistance when standing for 10 s. with one foot behind the other)	[No = 0, Yes = 1]	23.	Have you heart murmur?	[No = 0, Yes = 1]
9.	Bathing (Do you need assistance when bathing?)	[No = 0, Yes = 1]	24.	Do you have heart problem?	[No = 0, Yes = 1]
10.	BMI	[Normal = 0, Moderate = 0.50, Severe = 1]	25.	Do you have kidney diseases?	[No = 0, Yes = 1]
11.	Do you have bronchitis?	[No = 0, Yes = 1]	26.	Do you have liver diseases?	[No = 0, Yes = 1]
12.	Stand-ups from chair without using arms	[No = 0, Yes = 1]	27.	Do you have osteoporosis problem?	[No = 0, Yes = 1]
13.	Do you have arthritis?	[No = 0, Yes = 1]	28.	Have you had a Seizure?	[No = 0, Yes = 1]
14.	Do you need help to others when using toilet?	[No = 0, Yes = 1]	29.	Have you had a Stroke?	[No = 0, Yes = 1]
15.	Do you feel lonely?	[No = 0, Yes = 1]	30.	Have you urinary infection?	[No = 0, Yes = 1]

There was no missing value in the datasets and the calculated FI_30_ was a continuous score. Obviously, FI_30_ lies between 0 and 1, however, it was categorized, according to Clegg et al. (2016): FI_30_ ≤ 0.12 (No Frailty or Fit/Good Health); 0.12 < FI_30_ ≤ 0.24 (Mild Frailty/Slightly Poor Health); 0.24 < FI_30_ ≤ 0.36 (Moderate Frailty/Poor Health); and FI_30_ > 0.36 (Severe Frailty/Very Poor Health) for analysis purposes [40]. We further classified the FI_30_ into: No Frailty/Fit (Fairly Healthy, FI ≤ 0.24); and Frailty (Medical Conditions, FI > 0.24) to perform the multivariable binary logistic regression.

### Data analysis

We employed frequency distribution to understand the participants’ demographics. The relationships between the participants’ frailty (physio-psychosocial health) and socio-demographic factors were tested using bivariate analysis. The Pearson chi-square test was performed to observe the significant association between FI_30_ and socio-demographic variables. Student t-test of Frailty Index was employed to assess the mean difference between male and female older adults. The degree of associated risk factors was assessed by univariable [unadjusted odd ratio (unadjusted OR)] and multivariable odds ratios [adjusted odds ratio (adjusted OR)] obtained from the binary logistic regression model [41], where FI_30_ was a binary dependent variable, and physio-psychosocial variables were independent variables. This project’s data management and statistical analyses were carried out through IBM SPSS Statistics 20.0.

To ensure reliability and validity of the study results, we used a number of techniques: (i) employ SPSS 20.0 in data analysis; (ii) explore descriptive statistics of the sample (socio-demographic items) and their means and standard deviations; (iii) reliability analyses of the data used in calculating FI_30_ (Cronbach’s alpha, the reliability coefficient values for the variables used in FI_30_ is found 0.70); and (iv) use of two statistical techniques, namely chi-square test and logistic regression [42].

## Results

### Baseline demographics and prevalence of frailty

**Fig. 1:**
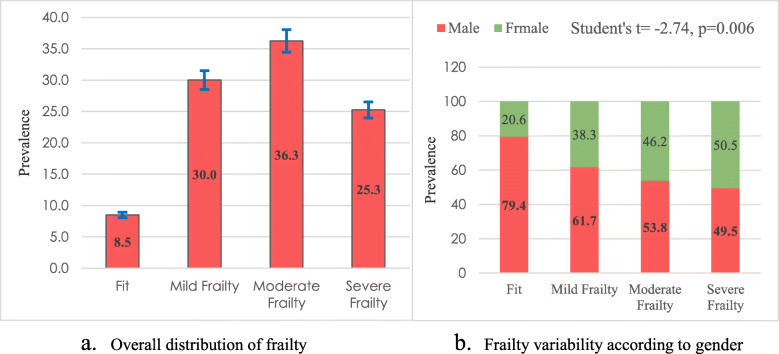
Distribution of the prevalence of elderly frailty with 95% confidence interval

The baseline demographics are presented in Fig. 1 and Table 3. Mean age of the participants was 63.61 (±8.73) years, with an age range of 55–100 years. More than half of them (57.3%) were male and illiteracy rate was 56.8%. One-third were engaged in different services and the rest of them were retired, housewife and small business with limited functional activities. Isolation because of divorce/widowed was identified as a strong predictor of elderly health (i.e. about 20% participants were divorced/widowed). Smoking habit was found in 27% participants.

**Table 3:** Association of FI_30_ with socio-demographic components

Characteristics	Frequency	Good Health/Fit [FI ≤ 0.12]	Slightly Poor Health/Mile Frailty [0.12 < FI ≤ 0.24]	Poor Health/Moderate Frailty [0.24 < FI ≤ 0.36]	Very Poor Health/Severe Frailty [FI > 0.36]	***P***-value
	n (%)	n (%)	n (%)	n (%)	n (%)	
**Age of the respondent**						
55–59	145(36.3)	22 (15.2)	51 (35.2)	53 (36.6)	19 (13.1)	0.0001
60–64	90 (22.5)	4 (4.4)	21 (23.3)	40 (44.4)	25 (27.8)	
65–69	66 (16.5)	4 (6.1)	30 (45.5)	19 (28.8)	13 (19.7)	
70+	99 (24.8)	4 (4.0)	18 (18.2)	33 (33.3)	44 (44.4)	
**Gender**						
Male	229 (57.3)	27 (11.8)	74 (32.3)	78 (34.1)	50 (21.8)	0.012
Female	171 (42.8)	7 (4.1)	46 (26.9)	67 (39.2)	51 (29.8)	
**Education**						
Illiterate	227 (56.8)	15 (6.6)	62 (27.3)	80 (35.2)	70 (30.8)	0.018
Literate	173 (43.2)	19 (11.0)	58 (33.5)	65 (37.6)	31 (17.9)	
**Occupation**						
Service	116 (29.0)	17 (14.7)	41 (35.3)	45 (38.8)	13 (11.2)	0.0001
Housewife/Others	284 (71.0)	17 (6.0)	79 (27.8)	100 (35.2)	88 (31.0)	
**Marital Status**						
Married	320 (80.0)	30 (9.4)	102 (31.9)	121 (37.8)	67 (20.9)	0.001
Widow/Divorce	80 (20.0)	4 (5.0)	18 (22.5)	24 (30.0)	34 (42.5)	
**Smoking Behavior**						
Non-Smoker	294 (73.5)	21 (7.1)	83 (28.2)	115 (39.1)	75 (25.5)	0.107
Smoker	106 (26.5)	13 (12.3)	37 (34.9)	30 (28.3)	26 (24.5)	
**Monthly Family Income in Taka (Dollars, considering $1 = 84.52 BDT, Accessed: October 06, 2020)**						
Less than 10,000 ($118.3)	118 (29.5)	3 (2.5)	32 (27.1)	44 (37.3)	39 (33.1)	0.020
10,000–20,000 ($118–$236.6)	139 (34.8)	16 (11.5)	41 (29.5)	51 (36.7)	31 (22.3)	
20,000–30,000 ($236.6–$354.9)	85 (21.2)	11 (12.9)	20 (23.5)	32 (37.6)	22 (25.9)	
30,000–40,000 ($354.9–$473.3)	31 (7.8)	3 (9.7)	12 (38.7)	11 (35.5)	5 (16.1)	
> 40,000 (>$473.3)	27 (6.8)	1 (3.7)	15 (55.6)	7 (25.9)	4 (14.8)	
**Family Types**						
Nuclear	172 (43.0)	14 (8.1)	58 (33.7)	57 (33.1)	43 (25.0)	0.522
Joint/Extended	228 (57.0)	20 (8.8)	62 (27.2)	88 (38.6)	58 (25.4)	
**Religion**						
Muslim	356 (89.0)	27 (7.6)	110 (30.9)	132 (37.1)	87 (24.4)	0.137
Non-Muslim	44 (11.0)	7 (15.9)	10 (22.7)	13 (29.5)	14 (31.8)	

The frailty index (FI_30_) ranged 0.05 to 0.71 where the mean FI_30_ was 0.28. Analysis of the health status reflecting on Frailty Index, most of the participants were observed in moderate frail or poor health (*n* = 145 (36.3%)) and 25.3% were severe frailty. Results also reported that maximum older adults (61.6%) were experiencing ‘poor to very poor’ health condition (moderate frailty to severe frailty), whereas only 8.5% were found fit or in good health (Fig. 1.a). Overall, the mean test (independent t-test) of the frailty index found a significant difference between male and female (mean FI of male: 0.271 (standard deviation: 0.118); mean FI of female: 0.303 (standard deviation: 0.114); *p* = 0.006). It was important to note that female older adults presented more health complexities with the increase of frailty severity and female older adults were severely frailty (50.5% vs 49.5%) compared with their male counterparts (Fig. 1.b).

### Association between FI_30_ and socio-demographic components

The association between older adults’ age groups and frailty was found statistically significant (*p* < 0.0001). Additionally, the participants’ gender, education, occupation, marital status and income were significantly (*p* < 0.05) associated with frailty (Table 3).

### Risk factors of the older adults’ frailty-related disorders

The physio-psychosocial risk factors of frailty were identified by unadjusted odds ratio and adjusted odds ratio with 95% confidence intervals after adjusting for a number of important covariates (Table 4). The participants who were of older age, female, were not involved with an occupation or activity outside the home and had low income were more likely to experience frailty severity than their counterparts. The older adults aged 60–64 years were significantly more frail (unadjusted odds ratio: 2.60, 95% CI: 1.63–4.12, *p* < 0.0001; Adjusted OR = 2.91, 95% CI: 1.61–5.23, *p* < 0.0001) than the pre-elderly age group (55–59 years), and the older adults aged 70 years and more, were 323% of a higher chance (unadjusted OR: 3.50, 95% CI: 2.18–5.62, p < 0.0001; Adjusted OR: 4.23, 95% CI: 2.26–7.92, p < 0.0001) of frailty (Medical Conditions) than the pre-elderly age group. Gender had a significant impact on frailty, where the risk of frailty severity was significantly higher (unadjusted OR: 2.226, 95% CI: 1.61–3.07, p < 0.0001; Adjusted OR = 1.487, 95% CI: 0.84–2.64, *p* < 0.0174) among the female than male older adults. Monthly income of the family was also a significant predictor of older adult’s frailty. The older adults from the higher income (Income>$473.3) family found a lower risk of frailty (unadjusted OR: 0.688, 95% CI: 0.32–1.48, *p* < 0.339; Adjusted OR: 0.294, 95% CI: 0.11–0.76, *p* < 0.011).

**Table 4:** Summary of Logistic Regression Model for Predicting Older Adults’ Health status through frailty

Characteristics	Unadjusted OR	CI	P-value	Adjusted OR	CI	P-value
**Age of the respondent**						
55–59	Ref	–	–	Ref	–	–
60–64	2.60	1.63–4.12	0.0001	2.910	1.61–5.23	0.0001
65–69	0.941	0.58–1.52	0.806	1.217	0.67–2.19	0.511
70+	3.500	2.18–5.62	0.0001	4.232	2.26–7.92	0.0001
**Gender**						
Male	Ref	–	–	Ref	–	–
Female	2.226	1.61–3.07	0.0001	1.487	0.84–2.64	0.0174
**Education**						
Illiterate	Ref	–	–	Ref	–	–
Literate	1.247	0.92–1.68	0.149	0.993	0.63–1.58	0.977
**Occupation**						
Service	Ref	–	–	Ref	–	–
Housewife/Others	1.958	1.53–2.50	0.0001	1.217	0.74–1.99	0.437
**Marital Status**						
Widow/Divorce	Ref	–	–	Ref	–	–
Married	2.636	1.61–4.31	0.0001	1.043	0.55–1.1.98	0.898
**Smoking Behavior**						
Non-Smoker	Ref	–	–	Ref	–	–
Smoker	1.20	0.76–1.64	0.560	0.732	0.437–1.23	0.236
**Monthly Family Income in Taka (Dollars, considering $1 = 84.52 BDT, Accessed: October 06, 2020)**						
Less than 10,000 ($118.3)	Ref	–	–	Ref	–	–
10,000–20,000 ($118–$236.6)	1.439	1.03–2.02	0.035	0.606	0.357–1.03	0.064
20,000–30,000 ($236.6–$354.9)	1.74	1.12–2.71	0.014	0.780	0.42–1.46	0.438
30,000–40,000 ($354.9–$473.3)	1.067	0.53–2.16	0.857	0.502	0.210–1.20	0.121
> 40,000 (>$473.3)	0.688	0.32–1.48	0.339	0.294	0.11–0.76	0.011
**Family Types**						
Nuclear	Ref	–	–	Ref	–	–
Joint/Extended	1.28	0.85–1.92	0.231	1.136	0.73–1.78	0.576
**Religion**						
Muslim	Ref					
Non-Muslim	0.994	0.52–1.89	0.98	0.791	0.38–1.64	0.527

## Discussion

We investigated, using the Frailty Index (FI_30_), the prevalence of frailty or physio-psychosocial health in Bangladeshi community-dwelling older adults and explored the associated risk factors. We found that most people in older age groups were susceptible to frailty, and at higher risk of medical conditions than the pre-elderly age group, which is consistent with previous studies [1, 4, 27, 43, 44]. In South Asian Context, our study presented a high prevalence of frailty (61.6%) in older adults who are experiencing ‘poor-very poor’ health (moderate to severe frailty), and this prevalence is greater than other South Asian countries (India - 26%; Pakistan - 55.4%; Nepal – 46.2%; and Sri Lanka – 15.2%). It is interesting that the prevalence of intermediate or pre-frail in Pakistan and Sri Lanka is consistent with our study findings [14, 18, 20, 45].

We conducted this study in a regional area of Bangladesh and the Frailty Index used predicts physio-psychosocial health for all older adults’ age groups. Three studies were conducted in regional areas of Colombia (12.2%); Mexico (10.7%); and Turkey (7.1%), where low prevalence of frailty was evident compared to our study [46–48]. The reasons of such low prevalence are unknown. Such low prevalence could be because of methodological issues or participant’s recruitment process, as identified by Siriwardhana et al. (2018) that voluntary participation underestimates the true prevalence and participants aged 70 years and older are less likely participate [20]. In contrast, a large number of older adults living in a regional area participated in this study and our FI_30_ indices were effective in explaining their frailty and or health conditions. Health professionals may consider using the FI_30_ indices to develop and implement evidence-based public health programs and clinical decision making in geriatric care in regional areas of South Asia.

As suggested by existing literature, our study found frailty was associated with old age, mobility and functional status. Analysis of the association between age and frailty implies that frailty is a critical predictor for overall health and the well-being status of older adults [49–52]. According to Cloney et al. (2015), frailty metrics are effective to identify the older adult’s risk of health conditions and, similarly, this study suggests that elderly persons medical conditions are more likely to increase with the advancement of age [53]. The findings of this study are comparable to the existing literature that, as measured using diverse frailty index indicators, elderly health is not even in a good position in Bangladeshi older adults [22, 25, 43, 44]. Most older adults ‘60–69 years’ experience slightly poor to poor health, while the older adults aged 70 years and above are at risk of severe medical conditions. The implication of our findings is similar with studies conducted in Sri Lanka and Pakistan in elaborating that South Asian older adults aged 65 years and above, especially those who live in regional areas, require more medical care support as their mobility and functionality decline rapidly at this stage.

In this study, frailty indices consider various aspects of general health assessment including self-reported medical history, biomarkers and performance in daily activities. Gender was identified as a risk factor for predicting the older adults’ frailty [14, 27]. This study’s findings agree on the previous studies reporting that female older adults in low-income countries, South Asia in particular, are generally reluctant about their health complexities and do not take proper initiatives for health check-ups [27, 54–56]. A considerable inconsistency in the research outcomes is that female older adults who live without a partner are more inclined to suffer medical conditions [57–59]. Investigations also reported that frailty was severe for those who have a low activity status in predicting potential health conditions, which is consistent with our study’s results that frailty is associated with females who are less likely to engage in physical activities or exercise [43, 60–63].

The literature shows, as our study identifies that overall health and well-being status of the older adults is compromised with the presence of low family income; because, low family income prevents them to maintain a healthy lifestyle [43, 64–66]. About two-thirds of the world’s poorer older adults live In South Asia, and they suffer from basic humanitarian problems including hunger and under-nutrition, low consumption of food, lack of housing, inadequate healthcare and illiteracy [9, 67]. Under such circumstances, Bangladesh has the third largest population of poor older adults in the world, and about 63% of them are jobless and 15% are engaged in daily labour, resulting in a lack of income and savings. Health conditions of the older adults often become severe for those in poor living circumstances, with limited healthcare services and home care support [7, 68]. While other studies in South Asia did not identify the association between low family income and frailty, this study adds to the knowledge that family income is an important risk factor of frailty, therefore, suggesting strengthening the social safety nets for poor older adults.

We experienced few limitations related to this study: (i) this was cross-sectional research that carries the limitations of such studies as they have a lack of strength in cause-effect analysis; (ii) the study was conducted in one city of Bangladesh because of our limited funds; and (ii) health illiteracy of the participants contributed to a lengthy data collection period. However, we are planning to conduct a wide regional survey in our next project, which is approved by the University Research Centre (URC), SUST. Our next study could extensively investigate the clinical assessment outcomes in relation to older adult’s frailty. Investigations into the influence of frailty on chronic health conditions and medical outcomes are warranted.

## Conclusion

The Frailty Index not only predicts the older adults’ physical health, but also measures their health-related quality of life governed by socio-demographic factors. The study findings confirm that community-dwelling older adults live in north-eastern region of Bangladesh are at high risk of frailty with two-thirds inclined to medical conditions requiring acute care. Being aged and female with low-income are influential risk factors to frailty/poor health. Patient activation strategies and primary care interventions including peer-health education, aerobic exercise sessions and an interdisciplinary public healthcare approach should be undertaken, especially for the female and older adults living without a partner/spouse, to strengthen geriatric care support. The public health leaders should also take the initiative to engage the older adults in paid or voluntary activities. It is claimed by the authors that frailty and associated socio-demographic factors can underscore physio-psychosocial health, thus, the findings are beneficial for policymakers in developing and implementing public health interventions for the older adults in developing countries like Bangladesh.

## Data Availability

The datasets generated and/or analyzed during the current study are available from the corresponding author on reasonable request to meshbahur.rahman@brfbd.org

## Abbreviations

BMI: Body Mass Index
BP: Blood Pressure
RBS: Random Blood Sugar
FI_30_: Frailty Index with 30 indicators
GDS: Geriatric Depression Scale
HBP: High Blood Pressure
